# Supplementary material for: Visualization of π-hole in molecules by means of Kelvin probe force microscopy
Source: Nat Commun. 2023 Aug 16;14:4954. doi: 10.1038/s41467-023-40593-3 (PMC10432393; doi:10.1038/s41467-023-40593-3)
Supplement: Supplementary file 1 — Supplementary Information [file 41467_2023_40593_MOESM1_ESM.pdf]

Supplementary Information

Visualization of π-hole in molecules by means of Kelvin probe force microscopy

Mallada et al

This supplementary information contains the following contents:

- Supplementary Figures 1-12
- Supplementary Tables 1-3

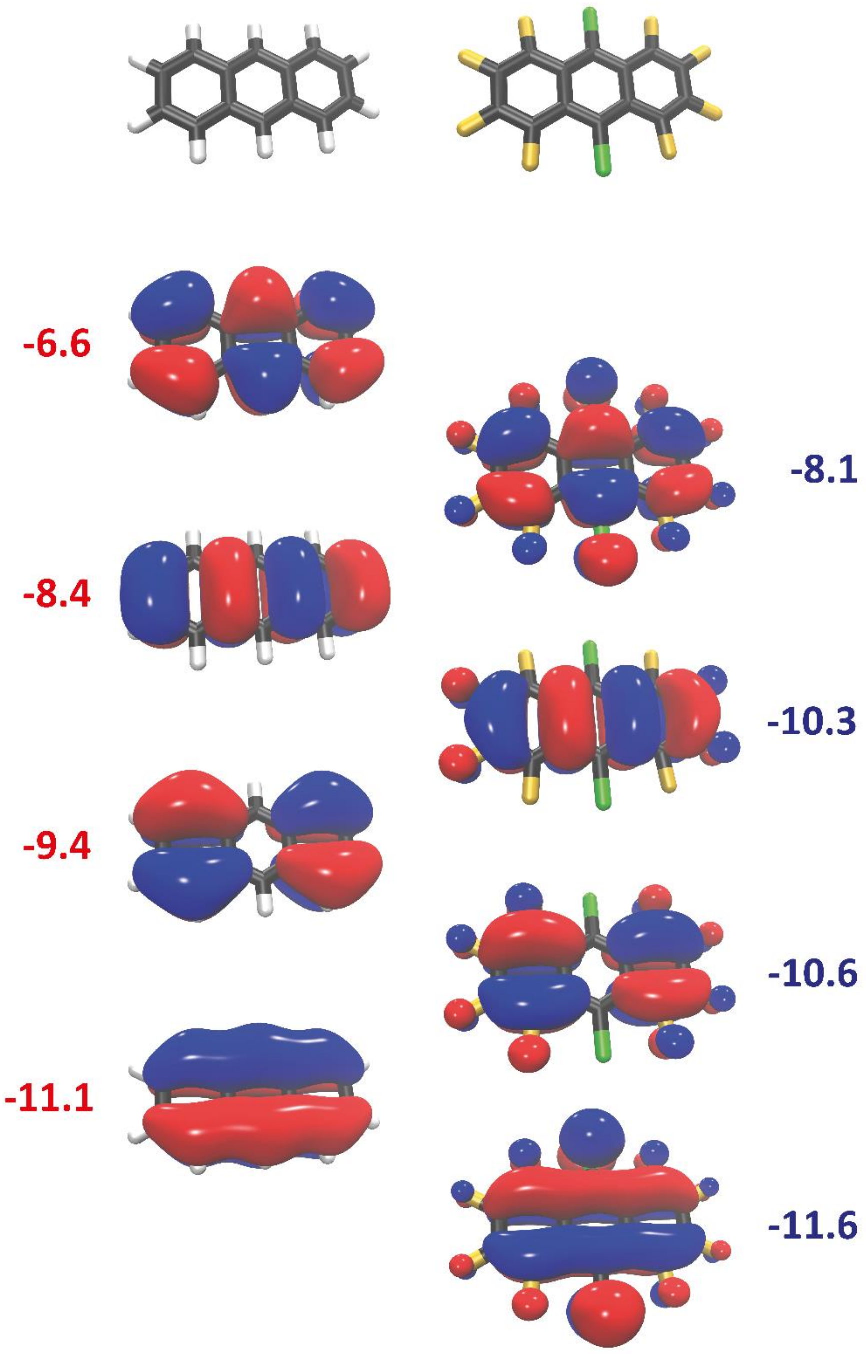

Supplementary Figure 1 | Frontiers orbitals HOMO, HOMO -1, HOMO -2 and HOMO -3 for free-standing An ($C_{14}H_{10}$, left) and FCl-An ($C_{14}F_8Cl_2$, right) with corresponding energies (eV) calculated at HF/cc-pVTZ level.

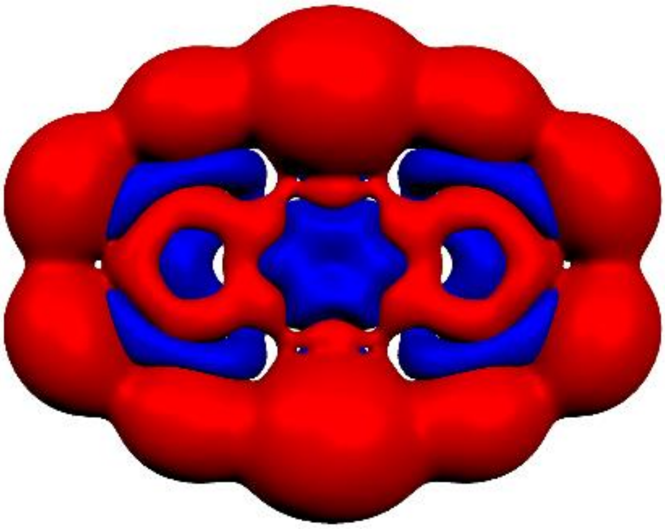

Supplementary Figure 2 | Electron density difference map of 9,10-dichlorooctafluoroanthracene versus anthracene [$\Delta\rho = \rho(C_{14}Cl_2F_8) - \rho(C_{14}H_{10})$]. Blue color shows decreasing of electron density, red color shows increasing of electron density.

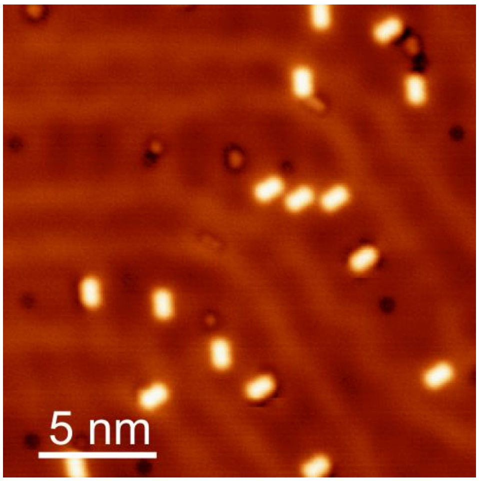

Supplementary Figure 3 | STM topography of single anthracene molecules on Au (111) (V_{Bias}=10 mV, I_{tunnel}=5 pA)

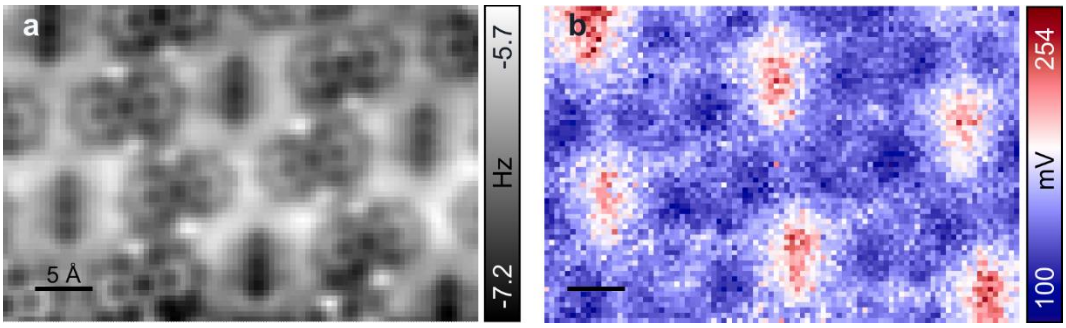

Supplementary Figure 4 | *Δf** and LCPD maps of an area including a full unit cell of the ordered anthracene and fluorinated anthracene assembly.

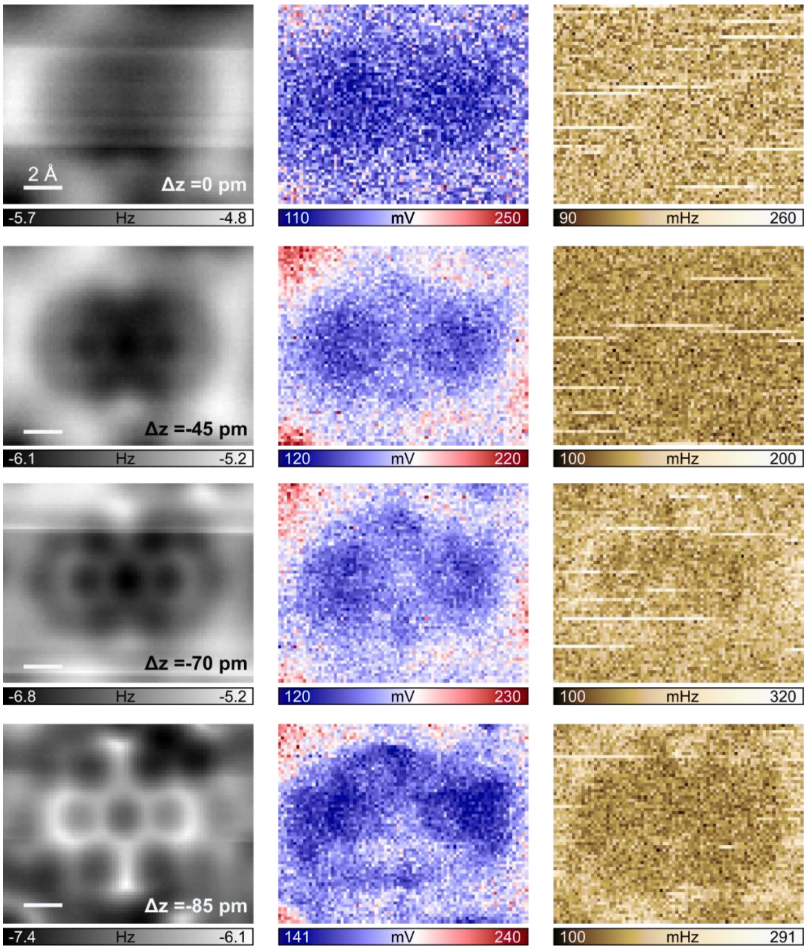

Supplementary Figure 5 | KPFM dataset from far-tip sample distances to close distances. First row displays a Δf * map, LCPD map and a RMS (root mean square) map at a tip-sample distance of z=+100 pm ($\Delta z = 0$ in this dataset) respect to the minima of interaction (see attractive region branch of the Δf-z spectroscopy on the central hexagon ring of FCl-An in **Figure.4**). The first column represents the Δf * parameter of the parabolic fit and provides information on the relative distance between the tip and an the FCl-An molecule. The second column displays the LCPD map, while the third column represents the average RMS fit residual of each KPFM curve. This map provides information of the parabolic fit's quality, as any systematic errors in the fit would be observed as distinct features in the RMS map.

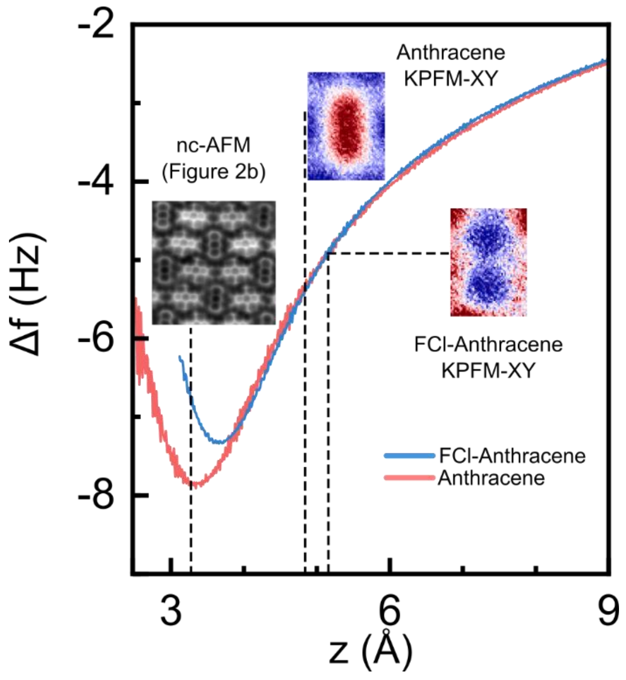

Supplementary Figure 6 | Relative heights of the LCPD maps. Point force spectroscopy measured on the central hexagon of anthracene (red) and fluorinated anthracene (blue) showing the relative z positions of the LCPD maps.

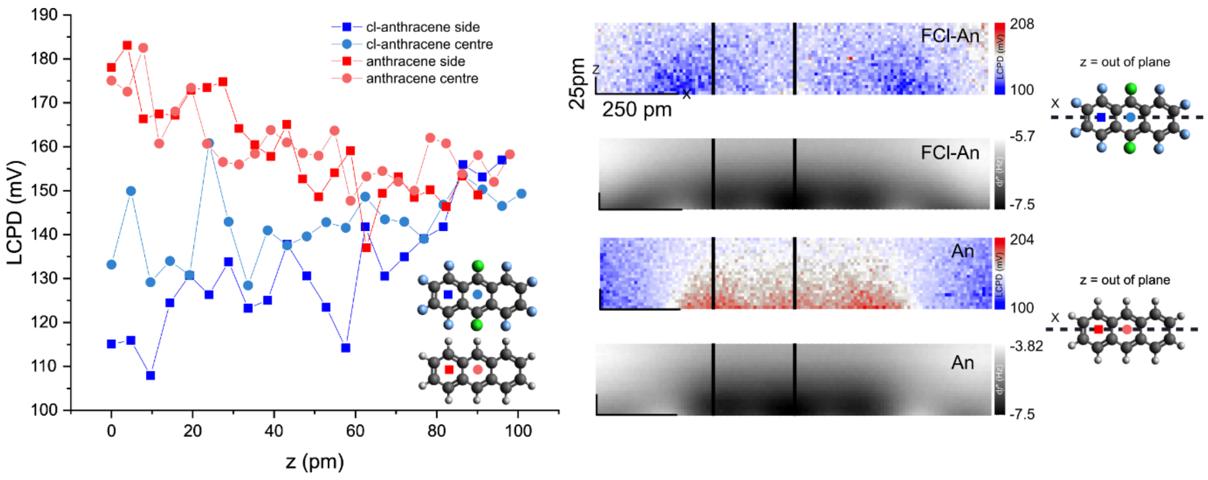

Supplementary Figure 7 | LCPD vs z on FCl-An and An. (Left) LCPD vs z over the left hexagon ring of FCl-An (blue squares), central hexagon (blue circles), and the left hexagon ring of An (red squares) and central hexagon (red circles). (Right) LCPD and $\Delta f*$ maps for FCl-An and An in the XZ plane (line over a molecule and perpendicular to the surface) obtained with a CO-tip showing the position of the LCPD(z).

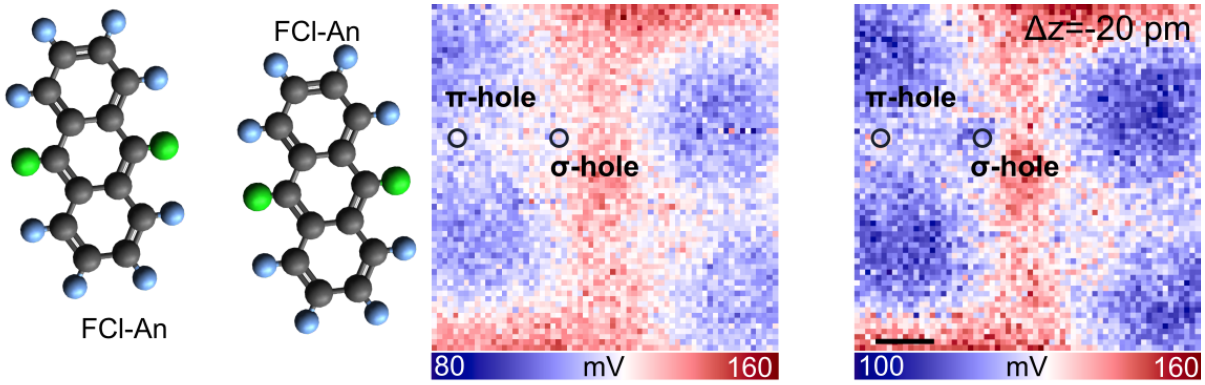

LCPD (mV)	far (Δz=0)	close (Δz=-20 pm)
π-hole	116 ± 5	112± 5
σ-hole	118± 5	118± 5

Supplementary Figure 8 | LCPD maps on two FCl-An molecules for a far (Δz =0 pm left LCPD map, +45 pm respect to the minima of interaction on the central hexagon as seen in **Fig. 4** or -35 pm respect a constant current STM setpoint of 10 mV, 5 pA) and close (Δz=-20 pm) tip-molecule distances. In both cases, the LCPD values on the Cl atoms, displaying a σ-hole, are similar to the measured LCPD of the π-hole within both FCl-An molecules.

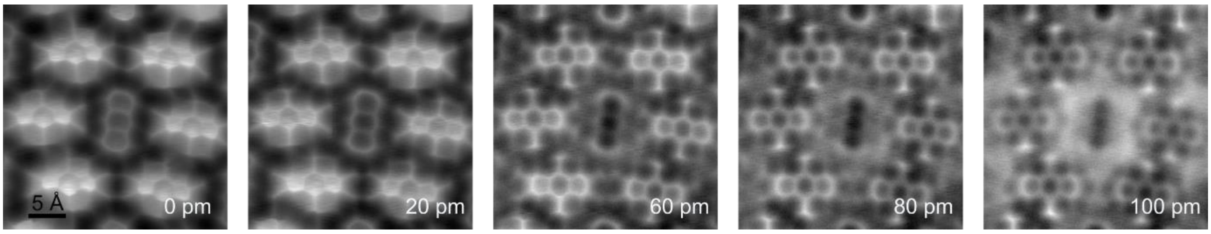

Supplementary Figure 9 | AFM vs z. Series of nc-AFM images with CO tip in constant height mode of the same area of the molecular assembly with tip-sample distance steps of Δz = 20 pm.

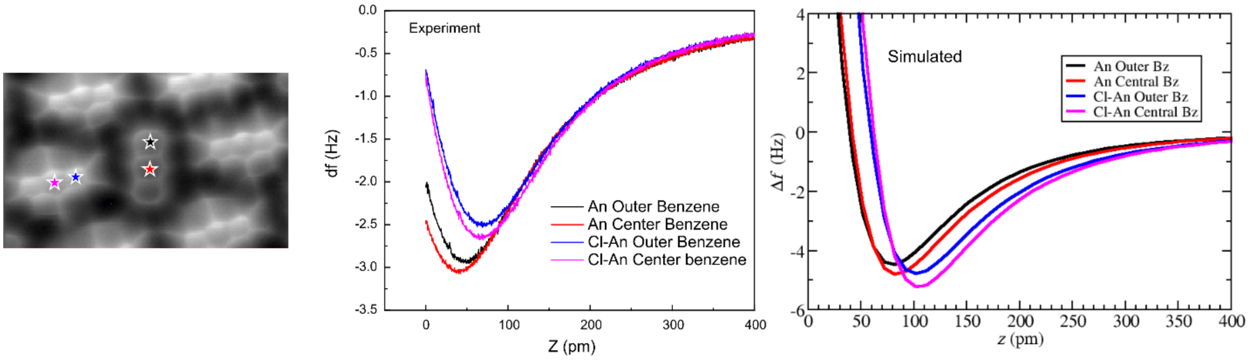

Supplementary Figure 10 | Experimental and PP-simulated Δ*f*(z) spectroscopies with CO-tip, over the central and outer rings of An (black and red solid lines) and the central and outer rings of FCl-An (purple and blue solid lines).

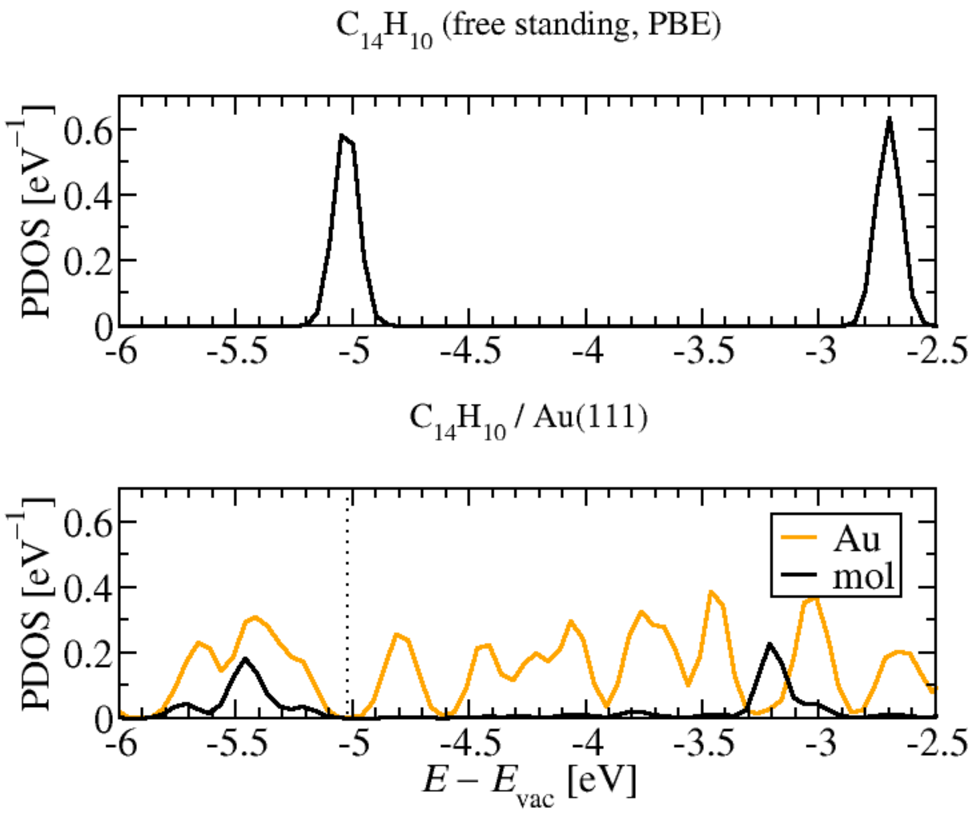

Supplementary Figure 11 | Projected density of states of free-standing $C_{14}H_{10}$ molecule and $C_{14}H_{10}$ on Au(111) surface revealing a negligible hybridization of frontiers HOMO and LUMO orbitals as well as a slight downshift of HOMO orbital from -5 eV (free-standing) to -5.4 eV on the Au(111) surface**.**

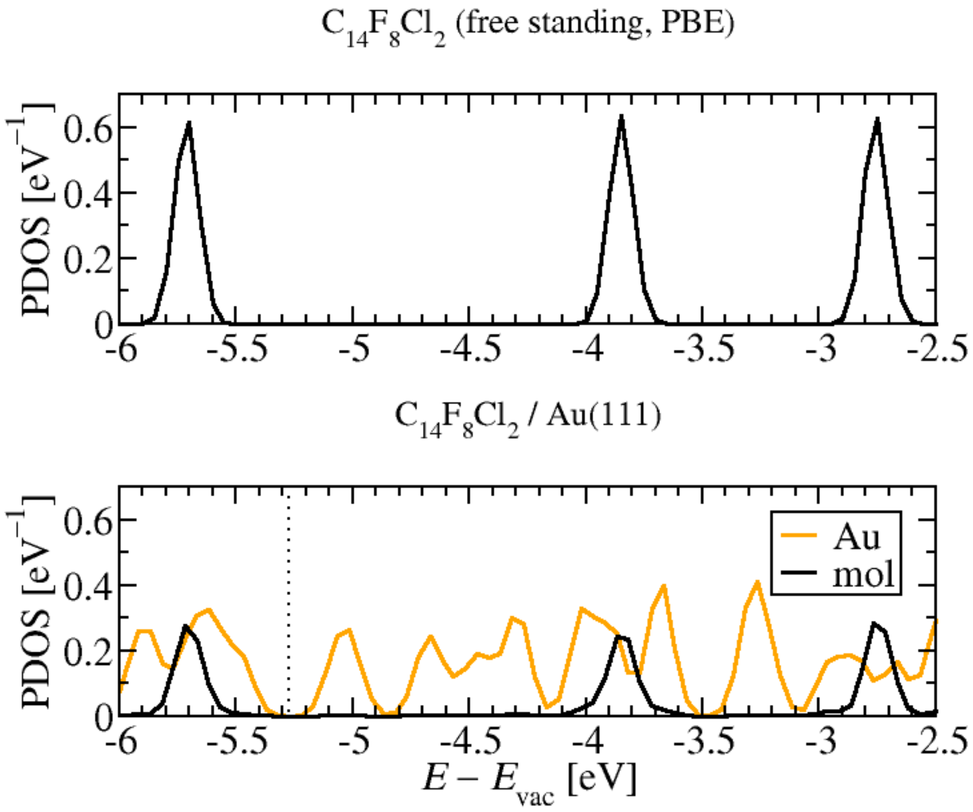

Supplementary Figure 12 | Projected density of states of free-standing $C_{14}F_8Cl_2$ molecule and $C_{14}F_8Cl_2$ on Au(111) surface revealing a negligible hybridization of frontiers HOMO and LUMO orbitals as well as a slight renormalization of molecular band gap upon adsorption on the Au(111) surface.

	DFT slab/ single molecule	DFT cluster/ single molecule	DFT slab/ Supramolecular assembly
$C_{14}H_{10}$/Au(111)	326	314	329
$C_{14}F_{10}$/Au(111)	347	320	-
$C_{14}Cl_2F_8$/Au(111)	340	321	349

Supplementary Table 1 | Adsorption height of $C_{14}H_{10}$,$C_{14}F_{10}$ and $C_{14}Cl_2F_8$ on Au(111) surface for different calculation models. The values of the adsorption height are provided in picometers.

	SAPT0 cluster	DFT cluster	DFT slab
$C_{14}H_{10}$/Au(111)	-50.50	-41.40	-39.63
$C_{14}F_{10}$/Au(111)	-42.18	-38.13	-41.23
$C_{14}Cl_2F_8$/Au(111)	-48.51	-42.12	-44.50

Supplementary Table 2 | Adsorption energies of $C_{14}H_{10}$, $C_{14}F_{10}$ and $C_{14}Cl_2F_8$ on Au(111) surface for different calculation models. The values of energy terms are provided in kcal/mol.

	Electrostatics	Exchange-repulsion	Induction	Dispersion	Total ΔE
$C_{14}H_{10}\cdots Au_{38}$	-43.02	84.09	-11.49	-80.08	-50.50
$C_{14}F_{10}\cdots Au_{38}$	-38.07	77.77	-7.98	-73.90	-42.18
$C_{14}Cl_2F_8\cdots Au_{38}$	-42.90	86.88	-8.50	-83.99	-48.51

Supplementary Table 3 | SAPT0 interaction energy decomposition for cluster models of $C_{14}H_{10}$,$C_{14}F_{10}$ and $C_{14}Cl_2F_8$ on Au(111) surface . The values of energy terms are provided in kcal/mol.
